# CSTAN: A Deepfake Detection Network with CST Attention for Superior Generalization

**DOI:** 10.3390/s24227101

**Published:** 2024-11-05

**Authors:** Rui Yang, Kang You, Cheng Pang, Xiaonan Luo, Rushi Lan

**Affiliations:** 1 Guangxi Key Laboratory of Image and Graphic Intelligent Processing, Guilin University of Electronic Technology, Guilin 541004, China; 1803304025@mails.guet.edu.cn (R.Y.); pangcheng3@mails.guet.edu.cn (C.P.); luoxn@guet.edu.cn (X.L.); 2 School of Computer Science and Information Security, Guilin University of Electronic Technology, Guilin 541004, China; 22032303032@mails.guet.edu.cn; 3 International Joint Research Laboratory of Spatio-Temporal Information and Intelligent Location Services, Guilin University of Electronic Technology, Guilin 541004, China

**Keywords:** deepfake detection, attention mechanism, detection model, feature extraction

## Abstract

With the advancement of deepfake forgery technology, highly realistic fake faces have posed serious security risks to sensor-based facial recognition systems. Recent deepfake detection models mainly use binary classification models based on deep learning. Despite achieving high detection accuracy on intra-datasets, these models lack generalization ability when applied to cross-datasets. We propose a deepfake detection model named Channel-Spatial-Triplet Attention Network (CSTAN), which focuses on the difference between real and fake features, thereby enhancing the generality of the detection model. To enhance the feature-learning ability of the model for image forgery regions, we have designed the Channel-Spatial-Triplet (CST) attention mechanism, which extracts subtle local information by capturing feature channels and the spatial correlation of three different scales. Additionally, we propose a novel feature extraction method, OD-ResNet-34, by embedding ODConv into the feature extraction network to enhance its dynamic adaptability to data features. Trained on the FF++ dataset and tested on the Celeb-DF-v1 and Celeb-DF-v2 datasets, the experimental results show that our model has stronger generalization ability in cross-datasets than similar models.

## 1. Introduction

With the development of deepfake technology, the forged images and videos it generates have brought various security risks to sensor systems. Highly realistic fake facial images or videos created by deepfakes may be used for identity forgery, allowing malicious individuals to deceive facial recognition systems and gain unauthorized access. Additionally, fabricated audio or video data may mislead sensors, making it difficult for them to distinguish between real and fake information, which could lead to incorrect security decisions by the system. The deepfake detection model is designed to process images captured by various sensors, such as cameras, to determine the authenticity of facial data. In practical applications, like intelligent surveillance and secure identification, the image data collected by sensor devices can be effectively analyzed by the deepfake detection model to detect forgeries, enhancing the security and robustness of the entire system.

In the field of deepfake detection, early research methods primarily relied on traditional image processing techniques. These models typically used basic image features as inputs, such as pixel values, edge information, and color histograms, and they performed binary classification by comparing the distinctive features between real and fake faces [1,2,3,4,5]. These early methods, which extracted simple visual features and constructed binary classification models, were able to detect fake faces to some extent. However, while these models performed well when forgery techniques were relatively primitive, they gradually exposed significant limitations as technology advanced.

With the development of Generative Adversarial Networks (GANs) [6], the quality of fake facial images has improved significantly, making fake images increasingly realistic. These advanced generation techniques have made fake images visually indistinguishable from real ones, thereby narrowing the gap between real and fake images. Conventional Convolutional Neural Network (CNN) [7] models tend to focus on specific forgery patterns or dataset characteristics, resulting in a sharp decline in detection capability when encountering novel forgery techniques or different types of fake images. This over-reliance on the features of the training data makes it difficult for models to handle the evolution and increasing complexity of forgery techniques, further reducing detection accuracy. To address this issue, scholars have proposed several innovative methods. These methods ingeniously focus on latent representations based on existing technologies, allowing the designed network models to accurately extract forged features from source faces. Zhou et al. [8] proposed a dual-stream deep network that effectively enhances deepfake detection performance by separately focusing on the visual appearance and local noise features of images. Similarly, Zhao et al. [9] designed a multi-attention detection network capable of capturing local features from specific regions of multiple faces, further enhancing the accuracy and robustness of the detection system. Inspired by this, Dong et al. [10], proposed a simple yet effective method named the ID-unaware deepfake detection model, to reduce the influence of implicit identity leakage. Furthermore, Huang [11] proposed a new implicit identity-driven face swapping detection framework, which shortens the distance between real faces and their explicit identities by designing Explicit Identity Contrast loss and Implicit Identity Exploration loss, while pushing fake samples away from their explicit identities. Chollet proposed the Xception architecture [12], which improves model parameter efficiency by replacing Inception modules with depthwise separable convolutions. Compared to Inception V3, Xception performs excellently on the ImageNet dataset and larger-scale datasets. This approach demonstrates the feasibility of enhancing the performance of classification models through a more streamlined and efficient convolutional structure, offering valuable insights for future network designs. Nguyen et al. introduced a multi-task learning framework [13], aimed at simultaneously detecting and segmenting manipulated facial images and videos. This method enhances performance by sharing information between tasks and employs a semi-supervised learning approach to improving model generalization. The network utilizes an encoder and a Y-shaped decoder, showing strong robustness against facial re-enactment and face-swapping attacks. By fine-tuning with a small amount of data, the model effectively addresses unseen attack types, providing a solution for data mismatch issues. Additionally, Li proposed a method based on detecting affine face-warping artifacts [14] that does not rely on deepfake-generated images as negative training samples. This method directly simulates artifacts, using simple image-processing operations, saving time and resources in data collection, while improving robustness in detecting forged videos from various sources. Wang et al. proposed the Multi-modal Multi-scale Transformer (M2TR) model [15], which detects local inconsistencies at different spatial scales and combines frequency and RGB information to enhance forgery detection accuracy. To further promote deepfake detection research, the authors introduced a high-quality deepfake dataset, SR-DF, and they experimentally demonstrated that the M2TR model significantly outperforms existing methods in detection effectiveness. Table 1 is a summary of the advantages and disadvantages of the above methods.

In summary, current advancements in deepfake detection have improved performance by utilizing efficient convolution operations, multi-task learning, affine artifact detection, and multi-modal fusion techniques. These innovations lay a solid foundation for future research in the field. However, these methods often rely on specific datasets, which limits their generalization. As deepfake generation techniques continue to evolve, the demand for higher security in sensor systems necessitates a focus on the fundamental differences between real and fake facial image features, to enhance the adaptability and robustness of detection models. Given that dynamic convolution can adaptively adjust the convolutional kernels for different inputs, allowing the network to adjust according to the varying characteristics of the input and, thus, reducing its reliance on specific training samples, and given that the attention mechanism enables the network to simultaneously focus on features at different scales, enhancing the model’s adaptability to diverse inputs, this paper presents a design for a deepfake detection model that combines dynamic convolutional networks and multi-dimensional attention mechanisms, aiming to improve the model’s generalization ability when facing different datasets.

**Figure 1:**
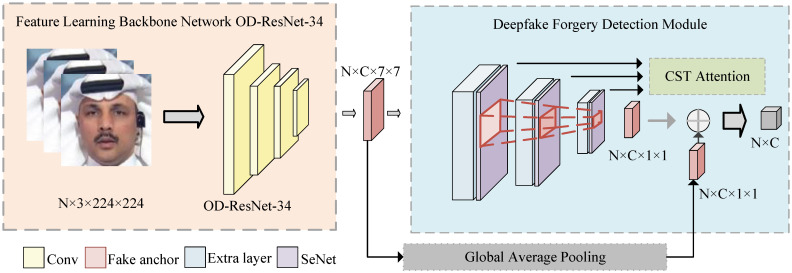
A deepfake forgery trace detection module with multi-attention mechanism fusion. The specific implementation of Fake anchor and Extra layer refer to DONG’S [10]. The input image size for the network is 224×224, where N represents the number of images. The backbone network employs OD-ResNet-34, and the output feature dimensions after processing through the backbone network are 7×7. Finally, the designed forgery detection module outputs a classification result of 1×1.

This paper proposes a deepfake detection network named CSTAN for superior generalization, which mainly consists of a designed feature-learning backbone network, OD-ResNet-34, and a forgery detection module with CST (Channel-Spatial-Triplet) attention, as shown in Figure 1. To enhance the dynamic adaptability of the backbone network to data characteristics, we designed OD-ResNet-34 by embedding ODConv into ResNet-34, thus improving the flexibility of the model in dealing with different datasets. To capture channel and spatial correlation, in order to obtain subtle local artifact information, we propose the CST attention mechanism in the forgery detection module, which enhances attention to forged regions by three different scales. Our experiment shows that enhancing the model’s attention to the forged regions can improve its generalization to various types of manipulated data. The main contributions of this paper are as follows:

- This paper presents a design for a deepfake detection model named CSTAN, with its core being the proposed CST multi-attention fusion mechanism, which can focus on local artifact regions to enhance the generalization capability of deepfake detection models.
- By introducing Omni-dimensional Dynamic Convolution (ODConv) into the feature-learning network, OD-ResNet-34 was developed, which enhances the adaptability of the image-feature-learning model to feature variations across different datasets, significantly improving deepfake detection accuracy.
- The experimental results indicate that, in comparison to multiple public models, CSTAN performs exceptionally well in cross-dataset detection tasks, validating its high adaptability and detection precision on unseen data.

## 2. Feature-Learning Backbone Network OD-ResNet-34

The designed feature-learning backbone network named OD-ResNet-34 was mainly designed by ODConv (Omni-dimensional Dynamic Convolution) [16] and ResNet-34 [17]. ResNet (Residual Network) is one of the most commonly used convolutional neural networks in the field of deep learning, particularly excelling in binary classification tasks. Proposed by Kaiming He and colleagues in 2015 [18], its innovative residual learning framework enables the effective training of deeper architectures, addressing the issues of gradient vanishing and performance degradation that arise as network depth increases. By introducing skip connections, ResNet allows gradients to flow directly through these connections, facilitating the propagation and learning of information. This design allows ResNet to achieve significant performance improvements across various binary classification tasks, such as image recognition, object detection, and speech classification. ResNet-18 and ResNet-34 were first trained on the FF++ dataset separately, then tested on the FF++ and Celeb-DF-v2 dataset for cross-dataset evaluation, as shown in Table 2. It can be observed that the performance of ResNet-34 was superior to that of ResNet-18. Therefore, ResNet-34 was chosen as the backbone network. ODConv amplifies the network’s representational prowess by introducing adaptable dynamic weights, allowing it to better capture complex features of input data. Dynamic convolution assists the network in better generalizing to unseen data, thus augmenting both the model’s generalization capacity and overall performance. Therefore, ODConv was used to improve the backbone network.

**Figure 2:**
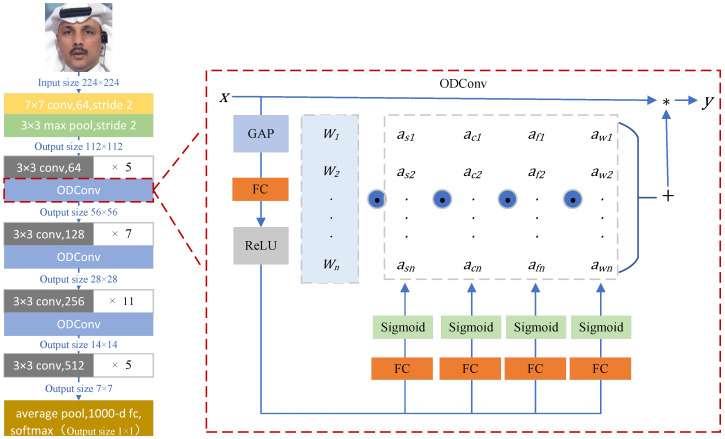
OD-ResNet-34 network. The number after the × sign represents the quantity of corresponding convolutional layers. For example, the first ×5 represents a convolution layer with a 3 × 3 kernel and 64 channels. There are five of them in the network. * is calculated as shown in Formula (1).

This study aimed to enhance the generalization capability of the model by introducing ODConv into ResNet-34. ODConv enhances the network’s representational ability by incorporating learnable dynamic weights, allowing it to better capture complex features within the input data. The dynamic convolution not only aids the network in generalizing better to unseen data, thus improving the model’s overall performance and generalization ability, but it also reduces the number of parameters through parameter sharing. This reduction in parameters lowers the model’s complexity and computational cost. The proposed OD-ResNet-34 is illustrated in Figure 2. First, the input layer receives an image of size 224×224. Next, the image passes through a 7×7 convolutional layer with 64 filters and a stride of 2, resulting in an output size of 112×112. Then, a 3×3 max pooling operation is performed with a stride of 2, maintaining an output size of 112×112 for the feature map. In the subsequent processing stage, the ODConv layer is introduced. Initially, a set of 3×3 convolution operations is conducted, using 64 filters, repeated 5 times, yielding an output size of 56×56. Following this, several groups of 3×3 convolution layers are employed for feature extraction, using 128 filters (repeated 7 times), 256 filters (repeated 11 times), and 512 filters (repeated 5 times), resulting in output sizes of 28×28, 14×14, and 7×7, respectively. Afterward, the network enters the Global Average Pooling (GAP) layer, which transforms the feature maps into a fixed-size output. Ultimately, this process outputs a feature vector of size 1×1, suitable for subsequent classification tasks.

ODConv replaces the original 3×3 convolutions in ResNet-34, enabling it to comprehensively capture features from the data, thereby demonstrating better adaptability to different input data. In the ODConv, the input feature vector *x* first undergoes a Channel-Wise Global Average Pooling (GAP) operation to compress it. Subsequently, the result is fed into a Fully Connected (FC) layer, where the compressed feature vector is mapped to a lower-dimensional space through the ReLU function. Following this, four branch heads are connected, with each branch corresponding to a Fully Connected layer and a Sigmoid function. These branches perform normalization operations, generating $\alpha_{si}$, $\alpha_{ci}$, $\alpha_{fi}$, and $\alpha_{wi}$, respectively. Along all four dimensions of the convolution kernel space $W_{i}$, four types of attention $\alpha_{si}$, $\alpha_{ci}$, $\alpha_{fi}$, and $\alpha_{wi}$ are computed. This enables ODConv to dynamically adjust finely across spatial size, input channel number, filter number (output channel number), and convolution kernel number. Such detailed adjustment allows OD-ResNet-34 to extract features more accurately, enhancing the learning and representation capability of the network. The process of feature maps through ODConv can be described by the following equation:

(1) $$y=(\alpha_{s1}⊙\alpha_{c1}⊙\alpha_{f1}⊙\alpha_{w1}⊙W_{1}+\ldots +\alpha_{sn}⊙\alpha_{cn}⊙\alpha_{fn}⊙\alpha_{wn}⊙W_{n})\times x$$

**Figure 3:**
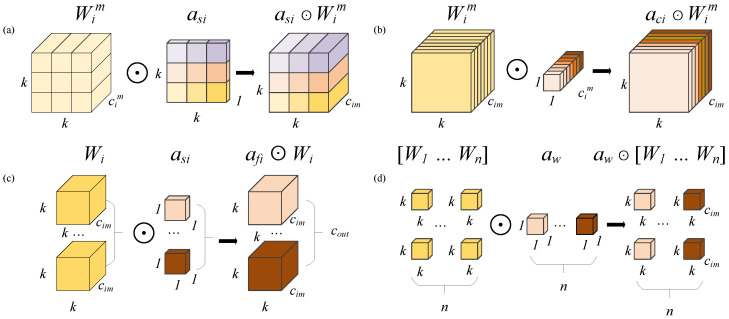
Attention computation across four dimensions: (**a**–**d**) represent the attention computation graphs for $\alpha_{si}$, $\alpha_{ci}$, $\alpha_{fi}$, and $\alpha_{wi}$, respectively.

In this context, $\alpha_{w1}$ represents the attention scalar of the convolution kernel $W_{i}$. The variables $\alpha_{si}\in R^{\left(k\times k\right)}$, $\alpha_{ci}\in R^{\left(c_{in}\right)}$, and $\alpha_{fi}\in R^{\left(c_{out}\right)}$ denote the three newly introduced attention mechanisms, corresponding to the spatial domain, input channel dimension, and output channel dimension, respectively. As shown in Figure 3, for the convolution kernel $W_{i}$, $\alpha_{si}$ assigns different attention weight values to the convolution parameters at the k×k spatial locations; $\alpha_{ci}$ assigns different attention weight values to the convolution filters of different input channels; $\alpha_{fi}$ assigns different attention weight values to the convolution filters of different output channels; lastly, $\alpha_{wi}$ provides different attention values to the overall $W_{n}$ convolution kernels.

By integrating the attention of the convolution kernel $W_{i}$ across the dimensions of position, input channels, output channels, and the entire kernel, the backbone network is able to learn the feature differences across different dimensions, thereby enhancing its ability to capture the contextual information of the image features. The model first processes the input image features using Global Average Pooling (GAP) and activates the features through the ReLU activation function. Next, it utilizes Fully Connected (FC) layers combined with four different attention heads to generate various types of attention values, with dimensions of k×k, $c_{in}\times 1$, $c_{out}\times 1$, and n×1, respectively. These attention values, through adaptive adjustments of the multidimensional information of the features, effectively enhance the model’s ability to recognize realistic forgery samples, thus improving the performance of deepfake detection.

## 3. Deepfake Forgery Detection Module with CST Attention Mechanism

**Figure 4:**
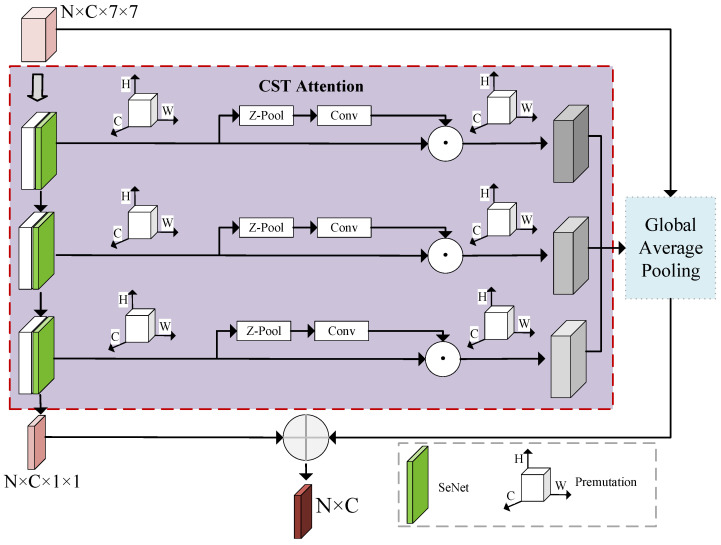
Deepfake forgery trace detection module with CST attention mechanism.

The fake faces created by current forgers are increasingly realistic, and conventional backbone networks are difficult to mine fake information. By integrating the channel attention and spatial attention of the feature map in two dimensions, the model can capture local fake details and improve detection accuracy by combining them with global context information. The designed deepfake detection module is mainly composed of the proposed CST attention. CST attention combines the advantages of both SENet (Squeeze-and-Excitation Network) [19] attention and Triplet attention [20], which extract subtle local information by capturing channel and spatial correlations, enhancing the attention to forged regions. The forgery detection module is illustrated in Figure 4.

In Figure 4, the aforementioned deepfake detection module, *N* and *C* represent the number of images and the number of channels, respectively. The image features extracted through the backbone network serve as input, and the model detects the locations of forged regions based on multi-scale anchor points. The implementation of multi-scale anchor point detection relies on adding four additional layers of different scales at the end of the backbone network, with feature map sizes progressively reduced to 7×7, 5×5, 3×3, and 1×1. In the final 1×1 layer, the feature maps are combined with the previous input through skip connections. This chapter adopts the method of incorporating the SENet attention mechanism in each additional layer, to accurately model the relationships between feature channels, establish interdependencies among channels, and perform adaptive recalibration of the channel feature responses, thereby improving detection accuracy. To further capture important information in the spatial dimension, this chapter introduces the Triplet attention mechanism. This mechanism is designed with a three-branch structure specifically to model the interaction features between spatial and channel dimensions. Each branch captures the relationships between different dimensions, aiming to strengthen the tight connections between input channels or spatial locations. Through this improvement, the model can comprehensively capture the cross-dimensional interaction information in the input data and calculate the corresponding attention weights. In the CST attention mechanism, each of the three branches establishes a close connection between the channel dimension *C* and the spatial height dimension *H*. For example, in the first branch, the input tensor *x* is rotated counterclockwise by 90° along the *H* axis to obtain the tensor $R^{\left(W\times H\times C\right)}$. After passing through the Z-Pool layer and Conv layer, the resulting tensor is $R^{\left(1\times H\times C\right)}$. The attention weight is generated using the Sigmoid function, which is then rotated clockwise by 90° along the *H* axis to ensure that the shape of the rotated feature map is consistent with the original input. This rotation process helps to enhance the correlation between channels and spatial positions from different perspectives, allowing the model to analyze cross-dimensional information in the feature map more comprehensively. The second and third branches operate similarly, ultimately averaging the tensors generated by the three branches C×H×W. This allows the model to effectively integrate information from various perspectives and dimensions, enhancing its comprehensive understanding of forged features. The fused results are fed into a Fully Connected layer, where further processing occurs to generate the final prediction output.

In the multi-scale detection module, with the addition of each layer a corresponding detector and classifier are designed. These detectors and classifiers are used to locate and classify anchor points in the forged images. Specifically, the detector is responsible for outputting the position offsets of each default anchor point, with a shape of N×4, where *N* represents the number of anchor points and 4 indicates the four coordinate parameters (such as the center point and width–height). The classifier is used to calculate the class confidence for each anchor point, with an output shape of N×2, where 2 represents the two classes: fake anchor points and real anchor points. If the Intersection over Union (IoU) between the anchor box and the ground truth of the forgery region exceeds a predetermined threshold, the default anchor box is labeled as “fake”, indicating it is related to the forged area. To enhance the model’s detection capability for the forgery region, this module establishes a short connection with the end of the backbone network through a 1×1 feature map. This short connection design helps to fuse global features from the backbone network with local features from the forgery detection module, thereby further enriching the forged face artifact features learned by the detection module. Finally, the output results fused through the short connection are passed to a Fully Connected layer for further processing, generating the final prediction result. This prediction result relies on the global loss function $L_{G}$ [10], which primarily includes the global classification loss $cls_{loss}$ and detection loss $det_{loss}$. By summing and weighting these losses, the overall loss function $L_{G}$ can be expressed as follows:

(2) $$L_{G}=\beta cls_{loss}+det_{loss}$$

where β is a positive coefficient that controls the balance between $cls_{loss}$ and $det_{loss}$. The $cls_{loss}$ measures the cross-entropy loss of the final prediction accuracy for classifying real and fake images. The $det_{loss}$ guides the learning of the deepfake detection module and includes the confidence loss $conf_{loss}$ and location loss $loc_{loss}$, which are used to evaluate the prediction results of each anchor, namely, whether they are true or fake anchors. The formula is as follows:

(3) $$det_{loss}=\frac{1}{N}\left(conf_{loss}\left(x,c\right)+\alpha \cdot loc_{loss}\left(x,l,g\right)\right)$$

where *N* is the data of the positive anchor boxes, specifically the forged face anchor boxes, x∈{0,1} is an indicator for matching the default anchor points with the true values of the detection region, *c* is the class confidence, *l* and *g* represent the predicted and true values from the deepfake detection module, respectively, and α denotes the positive weight.

By establishing stronger interactions between the channel and spatial dimensions, the CST attention mechanism can capture richer feature information, thereby enhancing the ability to recognize subtle forged features. The design of CST attention not only improves the model’s sensitivity to local details but also allows for more effective handling of complex forged features, further enhancing the model’s detection accuracy and stability. This multidimensional attention mechanism provides more refined feature extraction capabilities for the deepfake detection task, helping to address the challenges posed by various deepfake datasets.

**Figure 5:**
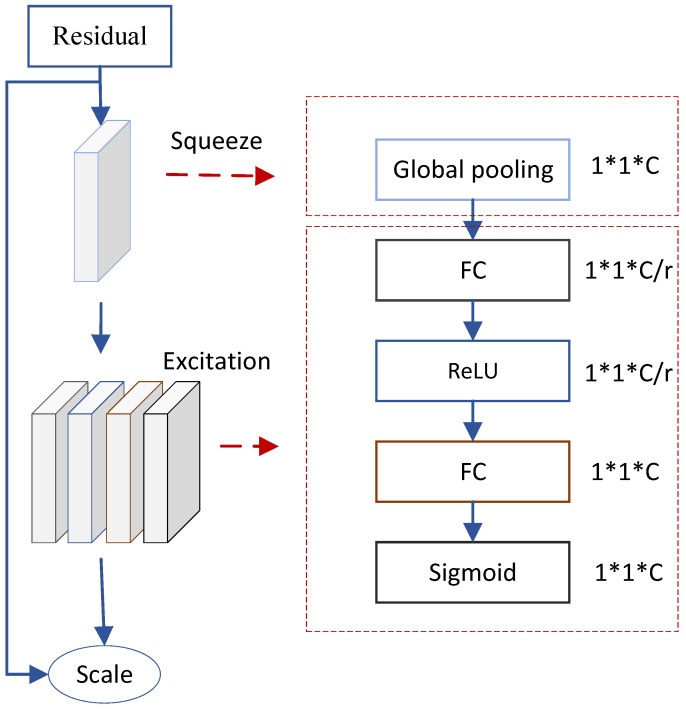
SENet structure. * is the common multiplication sign.

The SENet (Squeeze-and-Excitation Network) used in Figure 4 was proposed by Hu Jie and his research team in 2017 as a structure aimed at improving the performance of Convolutional Neural Networks. The SENet enhances network performance from the perspective of feature channels by embedding it into various neural network models. Its core idea is to learn the weights of each feature channel during the feature extraction phase, thereby suppressing features that are unrelated to the target task and enhancing the weights of feature channels that are relevant to the target task, thus achieving adaptive recalibration of feature channels. The main function of this mechanism is to utilize global information to optimize the selection of feature channels, enhancing the weight of useful features while reducing the influence of ineffective ones. The core operations of the SENet structure include two steps: Squeeze and Excitation, with the specific structure illustrated in Figure 5.

The design of the Squeeze operation is aimed at overcoming the limitations of convolutional kernels that operate solely within local receptive fields. Traditional convolution operations are unable to effectively capture the global relationships between channels, making it difficult for each convolution output unit to fully utilize contextual channel information. To address this issue, the Squeeze operation compresses the feature map into a vector of size 1×1×C through Global Pooling, where *C* represents the number of channels in the input feature map. This process condenses the global spatial information of the channels into a global feature, laying the groundwork for subsequent operations. The Excitation operation further processes this global feature, as illustrated in Figure 5. Compared to the Squeeze operation, the Excitation operation introduces three additional layers: two Fully Connected layers, one ReLU activation function, one Fully Connected layer, and a Sigmoid function. Through the two Fully Connected layers, feature information from various channels can be fused, and the Sigmoid function learns the nonlinear dependencies between channels. To reduce the model’s parameter count, the Excitation operation introduces a reduction factor *r* for channel dimensionality reduction. In the context of deepfake detection research, the value of *r* is set to 16 to effectively control the model’s complexity.

## 4. Experimental Environment

The FF++ [21] dataset was used as the training dataset, and the test datasets were 280 fake videos taken from subsets of FF++: FaceShifter, FaceSwap, Face2Face, Deepfakes, and NeuralTextures. Celeb-DF-V1 [22] contained 250 real YouTube videos and 795 fake videos. Celeb-DF-V2 [22] contained 300 real YouTube videos and 5639 fake videos.

The total number of epochs was 200, and each epoch had 512 randomly selected mini-batches. The initial learning rate was $3.6\times 10^{-4}$, which was reduced to $1\times 10^{-4}$ at epoch 10 and $5\times 10^{-5}$ at epoch 20. In the loss function, $cls_{loss}$ and $det_{loss}$ were set to 1 and 0.1 by default. The experimental hardware environment utilized an Intel(R) Xeon(R) W-2265 CPU (3.50 GHz) and an NVIDIA RTX A4000 GPU with 16 GB of memory, both sourced from Intel Corporation (Santa Clara, CA, USA) and NVIDIA Corporation (Santa Clara, CA, USA), respectively. The software environment consisted of the Ubuntu 16.04 operating system, the Python 3.10 programming language, and the PyTorch 1.10.0 deep learning framework. The performance of the model was evaluated using the AUC (Area Under the Curve) metric.

## 5. Experimental Results

The ablation experiments were carried out on different improvement points of this paper, and the results are shown in Table 3.

The “Baseline” refers to the ID-unaware deepfake detection model proposed by Dong et al. [10] in 2023 at CVPR. The experimental results were trained on the FF++ dataset and the testing on the FF++, Celeb-DF-v1, and Celeb-DF-v2. Compared to the Baseline, the proposed improvements in this paper showed an increase in AUC. In the FF++ dataset, the accuracy achieved by the Baseline was 99.24%. After introducing the SENet, the accuracy improved to 99.42%, an increase of 0.18% compared to the Baseline. With the addition of the Triplet loss, the accuracy further rose to 99.63%, improving by 0.39% over the Baseline. When using ODConv, the accuracy reached 99.55%, which was an increase of 0.31% compared to the Baseline. When employing the CST attention mechanism, the performance significantly improved to 99.84%, an increase of 0.60% over the Baseline. Finally, the CSTAN model achieved an accuracy of 99.96% on the FF++ dataset, improving by 0.72% over the Baseline model.

In the Celeb-DF-V1 dataset, the Baseline accuracy was 90.27%. After adding the SENet, the accuracy increased to 92.12%, a rise of 1.85% compared to the Baseline. The introduction of the Triplet loss further raised the accuracy to 93.50%, improving by 3.23%. The improvement from ODConv was modest, increasing the accuracy by only 1.71% to 91.98%. After using the CST attention mechanism, the accuracy climbed to 93.75%, an increase of 3.48% over the Baseline. The CSTAN model achieved accuracy of 94.24% on this dataset, which was a 3.97% improvement compared to the Baseline.

In the Celeb-DF-V2 dataset, the Baseline model’s accuracy was 86.95%. The SENet raised the accuracy to 88.12%, an increase of 1.17%. The introduction of the Triplet loss brought the accuracy up to 89.39%, improving by 2.44%. ODConv increased the accuracy by 1.46% compared to the Baseline, reaching 88.41%. The CST attention mechanism enhanced the performance to 90.56%, an improvement of 3.61%. The CSTAN model performed best, achieving accuracy of 91.75%, which was a 4.80% increase over the Baseline. The results of “Ours” demonstrate the effectiveness of the proposed model in cross-dataset testing.

To demonstrate the effectiveness of our model, we compared it with several state-of-the-art deepfake detection models. The results are presented in Table 4. Compared to the Multi-task (2019), Xception (2019), DSP-FWA (2018), M2TR (2022), CADDM (2023), and Deepfake Catcher (2024) models, respectively, based on evaluations within the FF++ dataset and across datasets using Celeb-DF-V2 dataset, it can be observed that our model demonstrated superior AUC compared to the other models overall, exhibiting better performance than the alternatives.

Table 4 shows the performance of different models on the FF++ and Celeb-DF-V2 datasets. It can be seen from the data that most of the models performed better on the F++ dataset, while the differences were more significant on the Celeb-DF-V2 dataset. In the F++ dataset, the Multi-task model performed the worst, with accuracy of 76.30%. The CSTAN model we designed achieved the highest score, 99.96%, on the F++ dataset, which was 23.66% higher than the Multi-task model, and it was the best model on this dataset. In the Celeb-DF-V2 dataset, the performance gap between the models was large. The accuracy of the Multi-task model was only 54.30%, which was the worst performance. However, the Xception model performed poorly on this dataset, only 49.03%, which was 5.27% lower than the Multi-task model. The PCL model reached 90.03%. The CSTAN model designed in this paper reached 91.75% on Celeb-DF-V2, which was 37.45% higher than the Multi-task model and 21.4% higher than the Deepfake Catcher model published in 2024. Our model was the best performing model in the table.

## 6. Model Analysis

**Figure 6:**
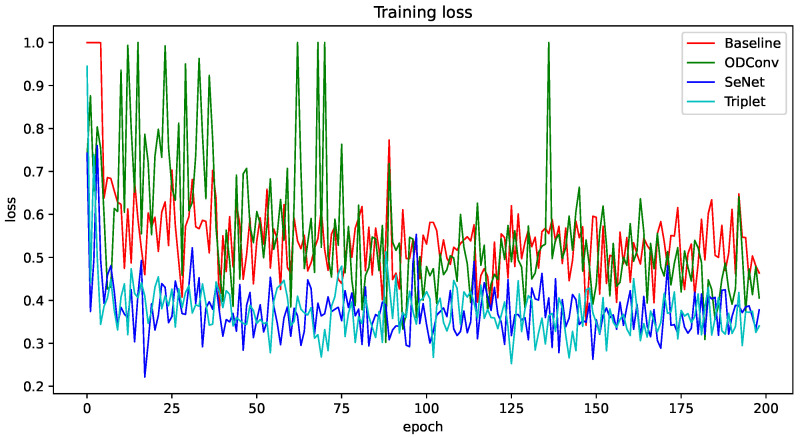
Plot of loss convergence for different improvement points.

**Figure 7:**
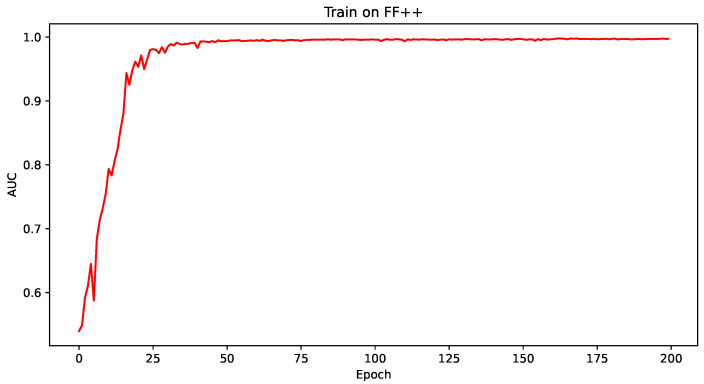
AUC plot obtained from training for 200 epochs on the CSTAN model for the FF++ dataset.

To validate the effectiveness of each improvement method, “Baseline”, “+SENet”, “+Triplet”, and “+ODConv” were trained on the FF++ dataset for 200 epochs, as shown in Figure 6. Each improvement point could stably converge in 200 epochs. The CSTAN model Trained continued for 200 epochs based on the pre-trained model DONG’s [10]; the AUC of its training process is shown in Figure 7.

**Figure 8:**
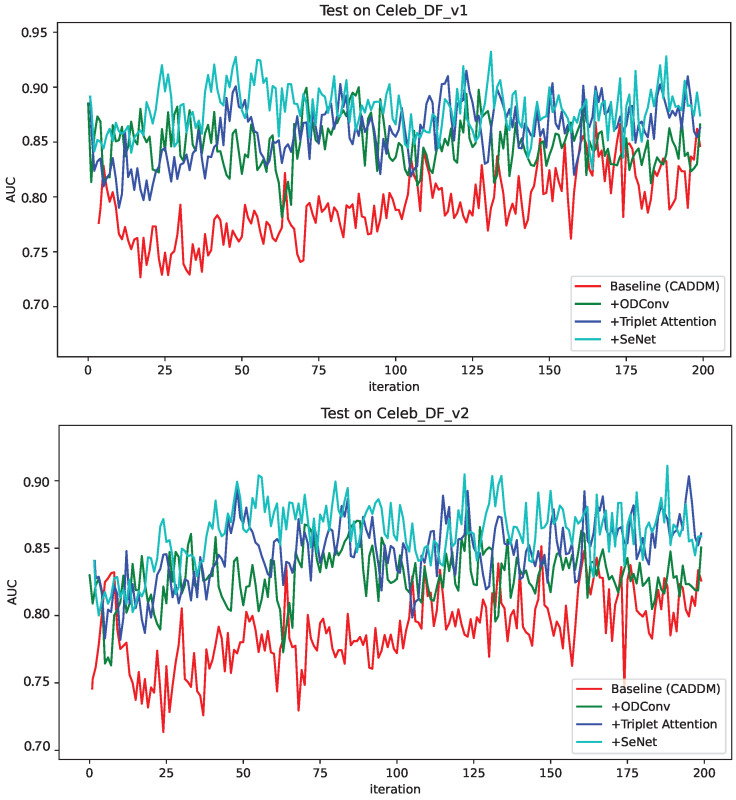
Randomized test results across datasets. The top figure shows the test results in the cycle of Celeb-DF-V1, and the bottom figure shows the test results in the cycle of Celeb-DF-V2.

To avoid model detection bias towards majority samples (e.g., Celeb-DF-V2, which contained 300 real YouTube videos and 5639 fake videos: if the model predicted the entire dataset as fake faces, the accuracy would have been as high as 0.94, which is obviously unreasonable), 100 real face videos and fake face videos in the Celeb-DF-V1 and Celeb-DF-V2 data were randomly selected for testing, and the test was repeated 200 times. The results are shown in Figure 8. It is obvious that different improvement points had different degrees of AUC improvement compared with the baseline.

## 7. Conclusions

This paper presents a new deepfake detection model named CSTAN, which mainly consists of the designed OD-ResNet-34 and CST attention. Through ablation experiments, the effectiveness of the model improvements was demonstrated. Furthermore, comparative experiments with other advanced deepfake detection models showed the significant advantages of our network in both cross-dataset and intra-dataset detection, proving our model’s ability to distinguish between real and fake facial information in sensor systems. Future research directions could include optimizing data augmentation strategies to enhance the model’s adaptability to different datasets, introducing more diverse and complex training data to improve model robustness, and exploring more effective transfer learning to enhance the model’s generalization across different datasets.

## Figures and Tables

**Table 1:** Summary of related models for deepfake detection.

Method	Advantages	Disadvantages
CNN [7]	Simple, effective	Poor novel forgery detection
Dual-stream deep network [8]	Enhanced performance	Limited forgery types
Multi-attention [9]	Accurate, robust	High resource demands
ID-unaware [10]	Reduces identity leakage	Limited adaptability
Face swapping [11]	Identity alignment	Large dataset required
Xception [12]	Parameter efficiency	Limited task customization
Multi-task learning [13]	Improved generalization	Increased complexity
Artifacts [14]	Resource-efficient	Misses some forgery types
M2TR [15]	Accurate inconsistency detection	High computational cost

**Table 2:** Performance comparison between ResNet-18 and ResNet-34.

Datasets	Resnet-18	Resnet-34
FF++	81.53	89.77
Celeb-DF-v2	56.88	67.22

The epochs for training are 200.

**Table 3:** Ablation experiment results (AUC): “+SENet” refers to the SENet structure utilized in the Baseline; “+Triplet” indicates the utilization of Triplet attention in the Baseline; “+ODConv” refers to the ODConv utilized in the Baseline; “Ours” represents the incorporation of all three methods on the Baseline.

Method	FF++	Celeb-DF-v1	Celeb-DF-v2
Baseline	99.24	90.27	86.95
ResNet-34 + SENet	99.42	92.12	88.12
ResNet-34 + Triplet	99.63	93.50	89.39
ResNet-34 + ODConv	99.55	91.98	88.41
ResNet-34 + CST	99.84	93.75	90.56
Ours (CSTAN)	99.96	94.24	91.75

**Table 4:** Public model comparison experiments results (AUC). The best experimental results are shown in bold.

Models	Backbone	FF++	Celeb-DF-v2
Multi-task [13]	∖	76.30	54.30
Xception [12]	Xception	99.58	49.03
DSP-FWA [14]	Resnet-50	93.00	64.60
M2TR [15]	Transformer	99.50	65.70
PCL [23]	ResNet-34	99.11	90.03
CADDM [10]	Resnet-34	99.24	86.95
Deepfake Catcher [24]	DenseNet-101	98.01	70.35
Ours (CSTAN)	Resnet-34	**99.96**	**91.75**

## Data Availability

The raw data supporting the conclusions of this article will be made available by the authors upon request.
